# Shikonin reduces tamoxifen resistance through long non-coding RNA uc.57

**DOI:** 10.18632/oncotarget.20809

**Published:** 2017-09-11

**Authors:** Chen-Han Zhang, Jue Wang, Lin-Xin Zhang, Yi-Han Lu, Tian-Hao Ji, Lu Xu, Li-Jun Ling

**Affiliations:** ^1^ Breast disease division, First Affiliated Hospital with Nanjing Medical University, Nanjing, Jiangsu Province, China

**Keywords:** shikonin, tamoxifen resistance, lncRNA, BCL11A, breast cancer

## Abstract

Tamoxifen resistance is a serious problem in the endocrine therapy of breast cancer. Long non-coding RNAs play important roles in tumor development. In this study, we revealed the involvement of lncRNA uc.57 and its downstream gene BCL11A in TAM resistance. Tamoxifen-resistant MCF-7R cells showed lower expression of uc.57 and higher expression of BCL11A mRNA and protein than the parental MCF-7 cells. Moreover, levels of uc.57 mRNA were lower and BCL11A mRNA were higher in breast cancer tissues than in precancerous breast tissues. Shikonin treatment reduced tamoxifen resistance in MCF-7R cells both *in vitro* and *in vivo*, targeting uc.57/BCL11A. Fluorescence *in situ* hybridization and RNA immunoprecipitation analyses showed that uc.57 binds to BCL11A. Uc.57 overexpression downregulated BCL11A and reduced tamoxifen resistance in MCF-7R cells both *in vitro* and *in vivo*. BCL11A knockdown also reduced tamoxifen resistance by inhibiting PI3K/AKT and MAPK signaling pathways. It thus appears shikonin reduces tamoxifen resistance of MCF-7R breast cancer cells by inducing uc.57, which downregulates BCL11A to inhibit PI3K/AKT and MAPK signaling pathways.

## INTRODUCTION

Breast cancer is the most common malignancy and second leading cause of cancer-related deaths in women worldwide [1]. Tamoxifen (TAM) has been extensively used for endocrine therapy for over four decades to improve the survival of hormone receptor-positive breast cancer patients [2]. However, TAM resistance results in breast cancer recurrence and mortality in a large number of cases [3].

Shikonin (SK) is an active ingredient isolated from the Chinese herb, *Lithospermum erythrorhizon*, which has been used for thousands of years in traditional Chinese medicine. It exerts anti-inflammatory [4], wound healing [5], and anti-cancer [6, 7] effects. SK inhibits estrogen-dependent tumor cell growth and promotes the anti-estrogen effect of endocrine therapy in breast cancer [8, 9]. It modulates mitogen-activated protein kinase (MAPK) and phosphoinositide 3-kinase (PI3K) pathways to suppress growth and survival of the malignant tumor cells [7, 10]. MAPK and PI3K pathways are critical players in TAM resistance [11–13] and their inhibition promotes TAM sensitivity [14–16]. This suggests that SK would be therapeutically beneficial to reduce TAM resistance in breast cancer cells.

Long non-coding RNAs (lncRNAs) are non-protein encoding transcripts that are longer than 200 nucleotides that are involved in tumorigenesis by post-transcritpional regulation of oncogenes and tumor suppressors [17]. LncRNAs are involved in TAM resistance via numerous mechanisms in addition to estrogen receptor (ER) signaling [11, 18, 19]. Many lncRNAs are conserved in humans [20, 21] and have potential clinical applications in anti-tumor therapy [22].

In this study, several ultra-conserved lncRNAs were selected and screened in a TAM-resistant breast cancer cell line and its parental cell line. Then, after proving SK could reduce TAM resistance, the relationship between SK-induced TAM sensitivity and lncRNAs was studied *in vitro* and *in vivo*.

## RESULTS

### Uc.57 and BCL11A are associated with TAM resistance in breast cancer cells

**Figure 1:**
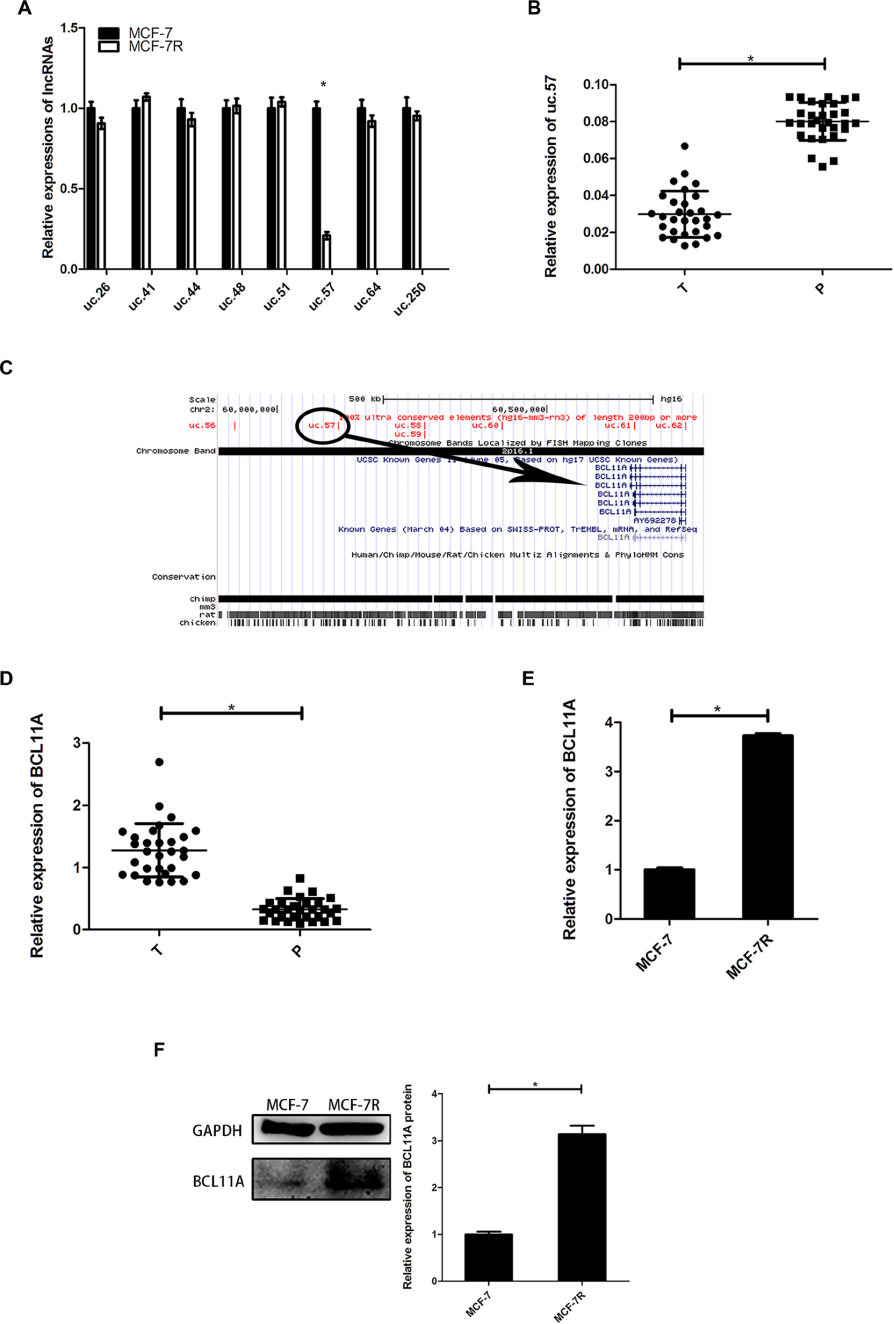
Expression of uc.57 and BCL11A correlates with TAM resistance in breast cancer (**A**) QRT-PCR analysis of 8 ultra-conserved lncRNAs, namely, uc.26, uc.41, uc.44, uc.48, uc.51, uc.57, uc.64, uc.250 in MCF-7 and MCF-7R cells. (**B**) QRT-PCR analysis of uc.57 expression in 30 paired breast cancer and precancerous tissues. (**C**) Schematic representation identifying *BCL11A* as the target gene of uc.57 in the UCSC genome database. (**D**) QRT-PCR analysis of BCL11A mRNA expression in 30 paired breast cancer and precancerous tissues. (**E**) QRT-PCR analysis of BCL11A mRNA expression in MCF-7 and MCF-7R cells. (**F**) Representative western blot showing BCL11A protein expression in MCF-7 and MCF-7R cells. Note: *denotes *P* < 0.05 compared to control; TAM, tamoxifen; SK, shikonin; MCF-7R, stable breast cancer cell line resistant to tamoxifen derived from MCF-7 cells.

We analyzed expression of ultra-conserved breast cancer-related lncRNAs (uc.26, uc.41, uc.44, uc.48, uc.51, uc.57, uc.64, uc.250) in MCF-7 and TAM-resistant MCF-7R cells. We observed low uc.57 expression in MCF-7R cells than in MCF-7 cells (Figure 1A). Moreover, uc.57 expression was lower in breast cancer tissues than in precancerous tissues (Figure 1B). This suggested that low uc.57 levels were linked to TAM resistance in breast cancer cells.

We analyzed the UCSC genome database and identified *BCL11A* as the uc.57 target gene on human chromosome 2 (Figure 1C). BCL11A expression was higher in breast cancer tissues than in precancerous tissues (Figure 1D). BCL11A mRNA and protein levels were detected in MCF-7R and MCF-7 cells. BCL11A was highly expressed in MCF-7R cells than in MCF-7 cells, which was inversely correlated with uc.57 expression (Figure 1E–1F). These data indicated that uc.57 and BCL11A were associated with TAM resistance.

### Shikonin reduces TAM resistance in breast cancer cells *in vitro* and *in vivo*

The IC_50_ for 4-OH-TAM in MCF-7 was 0.14 µM at 24 h (data not shown), but had no effect in MCF-7R cells. In MCF-7R cells, IC_50_ for SK was 2.55 µM at 24 h (data not shown), which was slightly lower than the dose selected for this study.

**Figure 2:**
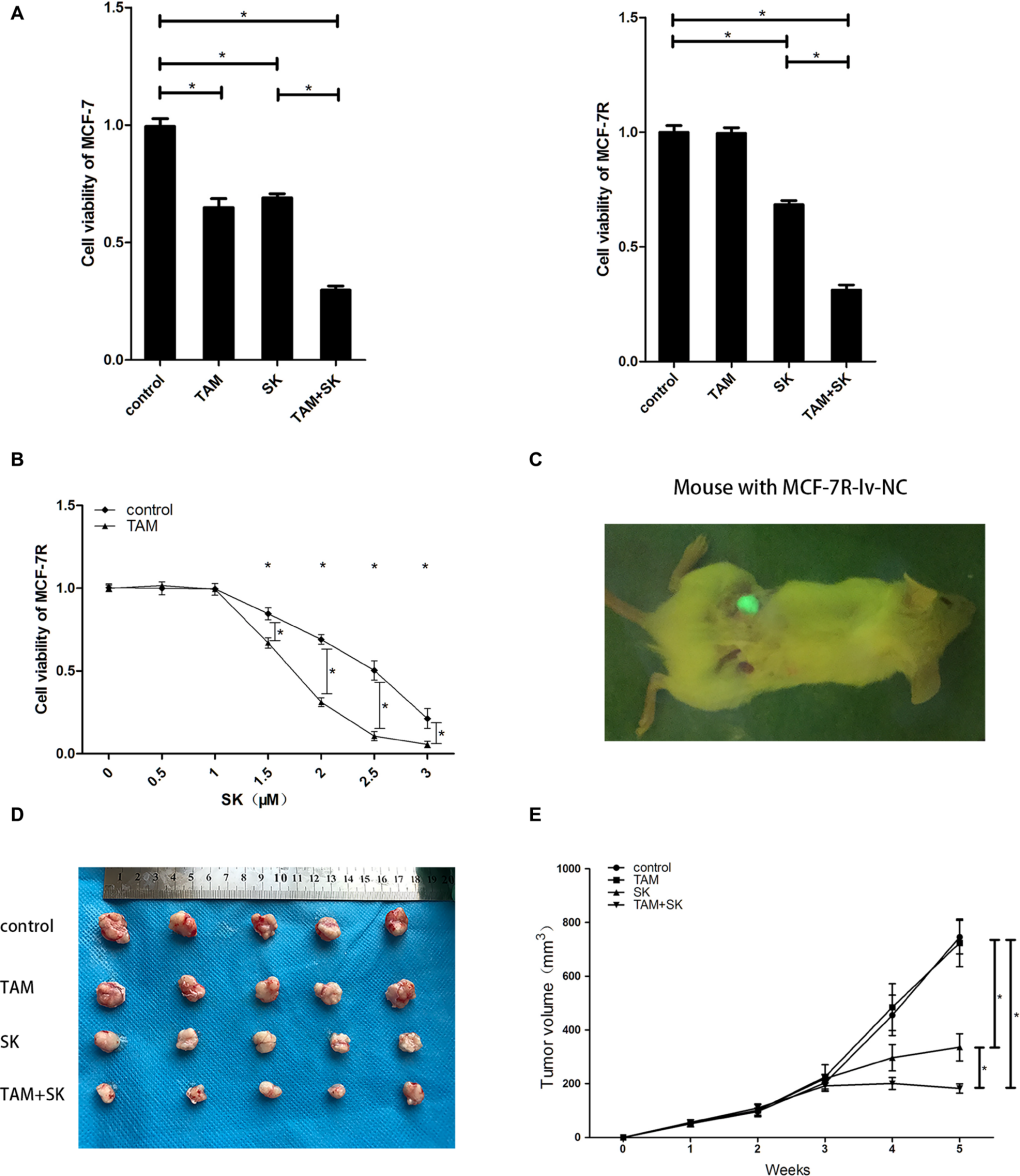
SK reduces TAM resistance in breast cancer cells *in vitro* and *in vivo* (**A**) CCK-8 assay showing viability of control, TAM, SK and TAM+SK treated MCF-7 and MCF-7R cells. (**B**) CCK-8 assay showing viability of MCF-7R cells treated with 0–3 µM SK with or without 0.1 µM TAM at 24 h. (**C**) Representative image showing tumors formed by MCF-7R-lv-NC cells transplanted in human breast tissue-derived SCID mice model. (**D**) Representative images of tumors at 5 weeks from control, TAM, SK and TAM+SK treated human breast tissue-derived SCID mice model with transplanted MCF-7R cells. (**E**) Tumor growth curves of control, TAM, SK and TAM+SK treated mice with MCF-7R cells transplanted in human breast tissue-derived SCID mice model. Note: *denotes *P* < 0.05 compared to control; MCF-7R-lv-NC, negative control MCF-7R cell line with green Fluorescence protein expression.

MCF-7 and MCF-7R cells were separately divided into the control (DMSO), TAM (0.1 µM 4-OH-TAM), SK (2 µM SK) and TAM+SK (0.1 µM 4-OH-TAM and 2 µM SK) treatment groups. After 24 h, cell viability was tested by CCK8 assay. The results revealed that in MCF-7 cells, both SK and TAM treatment can inhibit the cell growth and the co-treatment of SK and TAM was more effective than the single treatment of SK. In MCF-7R cells, TAM+SK treatment was most effective than SK treatment alone, whereas TAM treatment had no effect on viability of MCF-7R cells (Figure 2A). What’s more, TAM treatment inhibited approximately 35% cell growth of MCF-7 cell while SK+TAM treatment inhibited approximately 69% MCF-7R cell growth and 70% MCF-7 cell growth, which was about twice as much as the effect of TAM treatment on MCF-7 cells. We also treated MCF-7R cells with different doses of SK (0, 0.5, 1, 1.5, 2, 2.5, 3 µM) with or without TAM treatment (0.1 µM 4-OH-TAM) (Figure 2B). A lower dose of SK (0, 0.5, 1 µM) with or without TAM had no inhibitory effect on MCF-7R cells. A higher dose of SK (1.5, 2, 2.5, 3 µM) became effective to MCF-7R cells while a combination of such dose of SK and TAM inhibited the cell growth even more.

In the *in vivo* human breast tissue-derived SCID mice model (Figure 2C), we transplanted MCF-7R-lv-NC cells (negative control cells expressing GFP) and treated with control, TAM, SK or TAM+SK for 5 weeks. The tumor size of the TAM group was similar to control group, whereas tumor size was reduced in the SK group. Moreover, tumor volume was significantly reduced in TAM+SK group than in SK group alone (Figure 2D, 2E). Since TAM treatment alone had no effect on MCF-7R cells, these data suggested that SK reduced TAM resistance of MCF-7R cells in the TAM+SK group.

### SK decreases TAM resistance by inhibiting PI3K/AKT and MAPK pathways through uc.57/BCL11A

**Figure 3:**
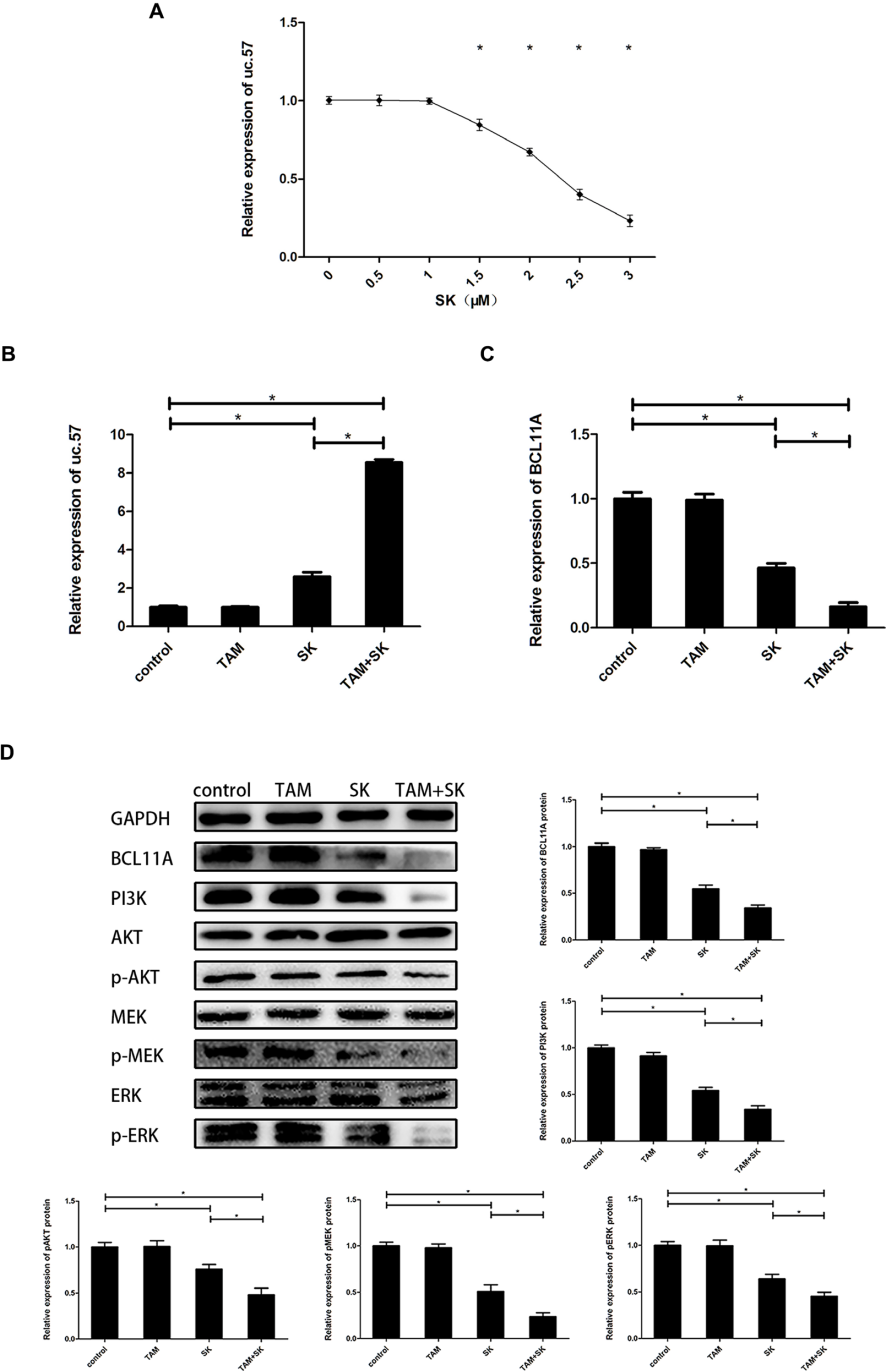
SK reduces TAM resistance of breast cancer cells by upregulating uc.57 and downregulating BCL11A and related PI3K/AKT and MAPK pathways (**A**) QRT-PCR analysis of uc.57 levels in MCF-7R cells treated with 0–3 µM SK. (**B**) QRT-PCR analysis of uc.57 expression in control, TAM, SK and SK+TAM treated MCF-7R cells. (**C**) QRT-PCR analysis of BCL11A mRNA expression in control, TAM, SK and SK+TAM treated MCF-7R cells. (**D**) Representative western blot showing expression of BCL11A, PI3K, AKT, p-AKT, MEK, p-MEK, ERK, p-ERK proteins in control, TAM, SK and SK+TAM treated MCF-7R cells. Note: *denotes *P* < 0.05 compared to control.

We observed a dose-dependent increase in uc.57 levels in MCF-7R cells treated with 0–3 µM SK (Figure 3A). Then, we determined expression of uc.57 and BCL11A in control, TAM, SK, and TAM+SK treated MCF-7R cells. While uc.57 levels in TAM and control groups were low, its expression increased in SK and TAM+SK treatment groups (Figure 3B). Conversely, BCL11A mRNA levels were high in control and TAM alone treated MCF-7R cells, but decreased in the SK and TAM+SK treatment groups (Figure 3C). Western blot analysis demonstrated that BCL11A protein expression was similar to its mRNA profile in the 4 treatment groups (Figure 3D). In addition, both SK and TAM+SK treatments downregulated PI3K/AKT and MAPK (MEK/ERK) signaling pathways (Figure 3D). These data suggested that SK inhibited PI3K/AKT and MAPK signaling pathways that promote TAM resistance by downregulating BCL11A, which was a result of uc.57 upregulation.

### Uc.57 negatively regulates BCL11A

**Figure 4:**
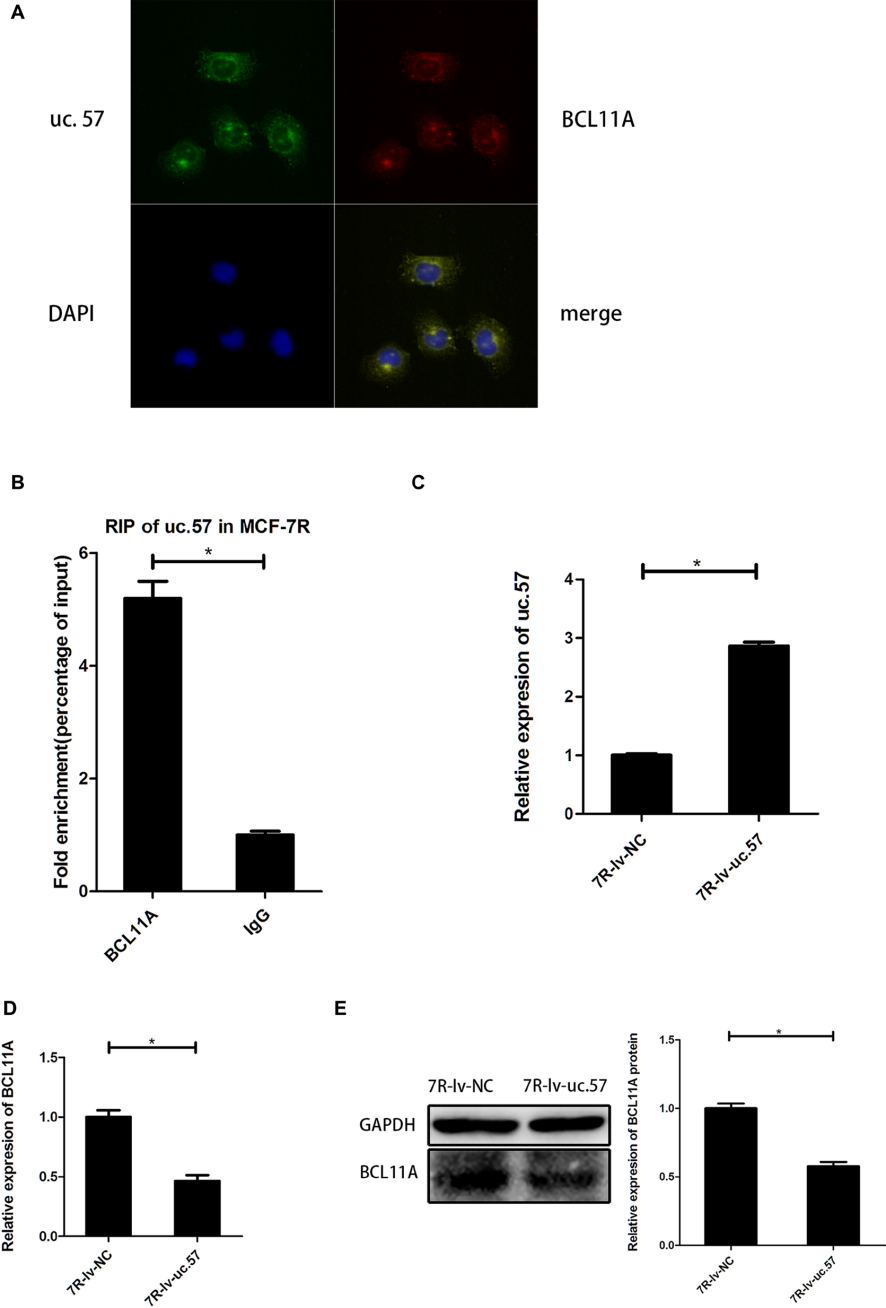
Uc.57 negatively regulates BCL11A expression in MCF-7R cells (**A**) FISH analysis showing interaction between lncRNA uc.57 and BCL11A mRNA. (**B**) RIP assay analysis showing that lncRNA uc.57 binds BCL11A in MCF-7R cells. The immunoprecipitated RNA was analyzed by qRT-PCR and normalized relative to input RNA and plotted as fold enrichment relative to IgG control. (**C**) QRT-PCR analysis of lncRNA uc.57 overexpression in MCF-7R-lv-uc.57 and MCF-7R-lv-NC cells. (**D**) QRT-PCR analysis of BCL11A mRNA expression in MCF-7R-lv-uc.57 and MCF-7R-lv-NC cells. (**E**) Representative western blot analysis showing BCL11A protein expression in MCF-7R-lv-uc.57 and MCF-7R-lv-NC cells. Note: *denotes *P* < 0.05 compared to control; MCF-7R-lv-NC denotes negative control MCF-7R cell line derived from MCF-7R cells; MCF-7R-lv-uc.57 denotes stable MCF-7R cell line overexpressing lncRNA uc.57.

We performed the FISH assay of uc.57 and BCL11A RNAs in MCF-7R cells and demonstrated that they co-localize with each other, mostly in the cytoplasm (Figure 4A). RIP assay demonstrated physical interaction between uc.57 and BCL11A in MCF-7R cells (Figure 4B). We then compared BCL11A mRNA and protein levels in uc.57 overexpressing MCF-7R-lv-uc.57 cells (Figure 4C) and MCF-7R-lv-NC control cells. We observed that overexpression of uc.57 downregulated BCL11A mRNA and protein levels (Figure 4D, 4E). These data suggested that uc.57 negatively regulates BCL11A.

### Uc.57 overexpression reduces TAM resistance *in vitro* and *in vivo*

**Figure 5:**
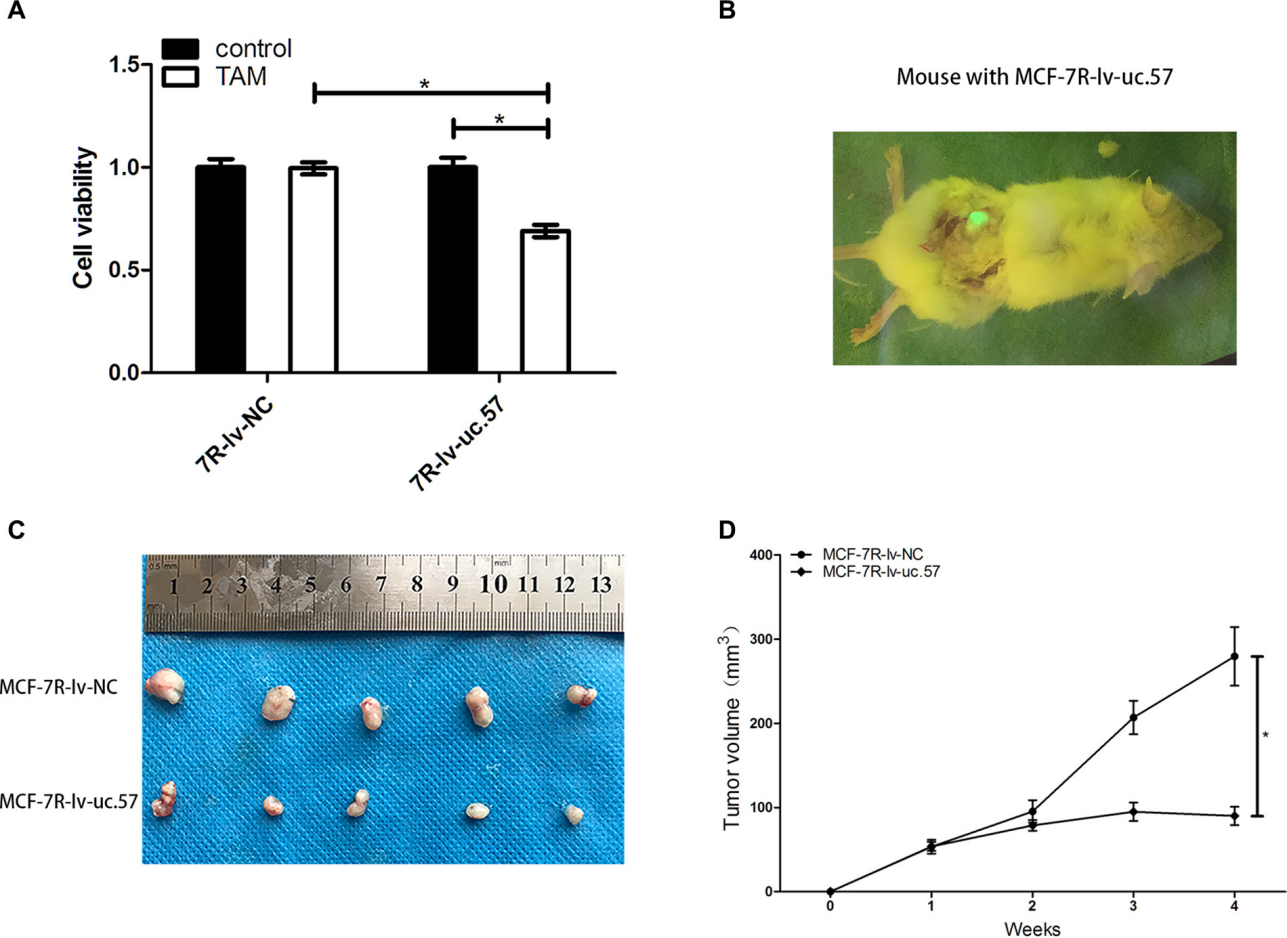
LncRNA uc.57 overexpression reduces *in vitro* and *in vivo* TAM resistance in MCF-7R cells (**A**) CCK8 assay showing cell viability of MCF-7R-lv-NC and MCF-7R-lv-uc.57 cells treated with or without 0.1 µM TAM. (**B**) Representative image showing tumors from xenografted MCF-7R-lv-uc.57 cells in human breast tissue-derived mouse model. (**C**) Representative images showing tumors from TAM treated mice xenografted with MCF-7R-lv-NC and MCF-7R-lv-uc.57 cells in human breast tissue-derived mouse model at 4 weeks after implantation. (**D**) Tumor growth curves of TAM treated mice xenografted with MCF-7R-lv-NC and MCF-7R-lv-uc.57 cells in human breast tissue-derived mouse model. Note: *denotes *P* < 0.05 compared to control.

We treated MCF-7R-lv-uc.57 and MCF-7R-lv-NC cell lines with 0.1 µM 4-OH-TAM and observed that uc.57 overexpressing MCF-7R-lv-uc.57 cells were sensitive to TAM, whereas MCF-7R-lv-NC cell line remained resistant to TAM (Figure 5A). We xenografted MCF-7R-lv-NC and MCF-7R-lv-uc.57 cells into human breast tissue-derived SCID mice model (Figure 5B). After four weeks of TAM treatment, we observed that the average tumor size from transplanted MCF-7R-lv-uc.57 cells was smaller than tumors derived from MCF-7R-lv-NC cells (Figures 5C, 5D). The data suggested that uc.57 overexpression reduces TAM resistance in MCF-7R cells, both *in vitro* and *in vivo*.

### BCL11A knockdown reduces TAM resistance by inhibiting PI3K/AKT and MAPK pathways

**Figure 6:**
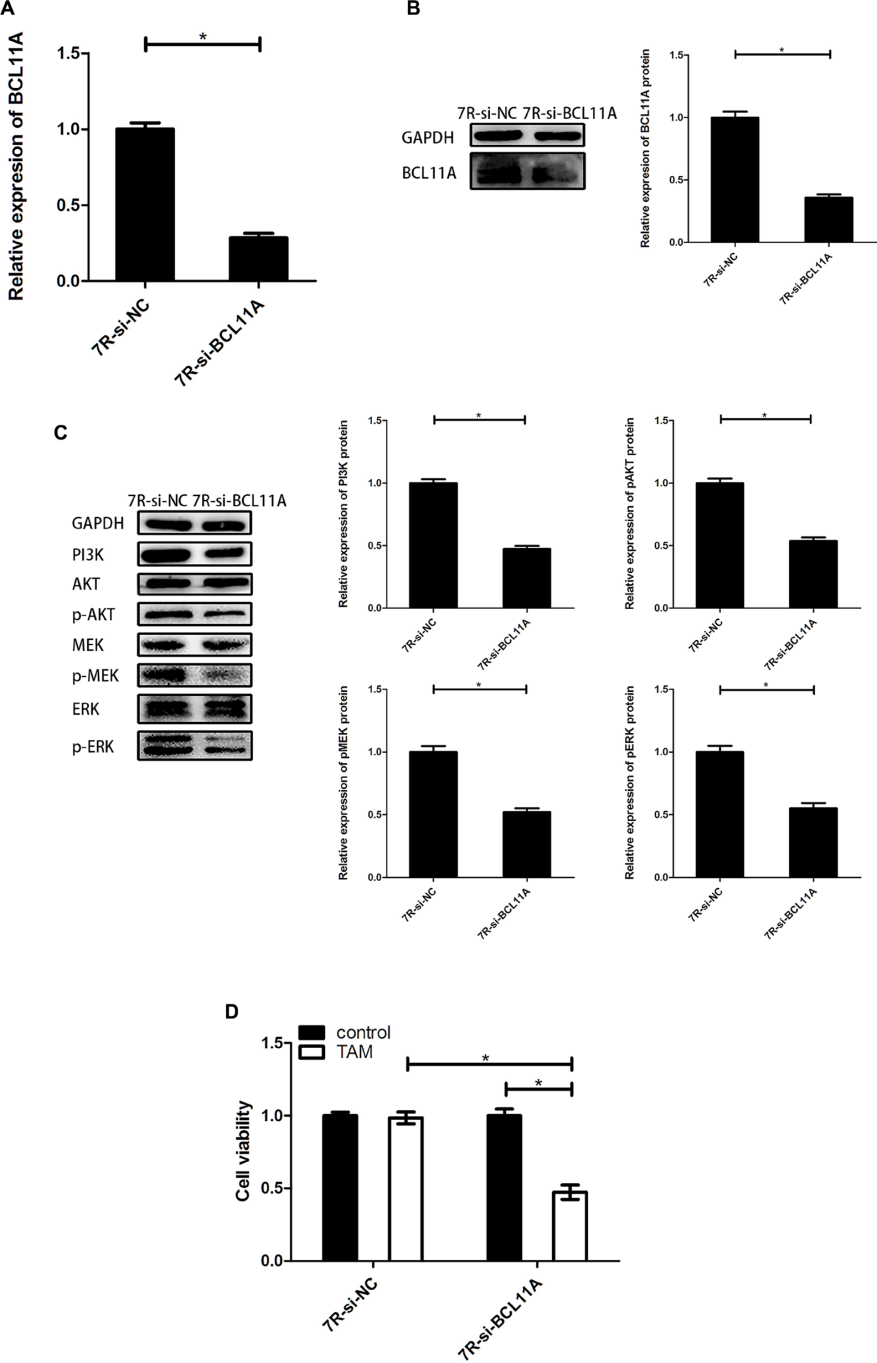
BCL11A knockdown reduces TAM resistance by inhibiting PI3K/AKT and MAPK signaling pathways (**A**) QRT-PCR analysis of BCL11A mRNA in MCF-7R-si-BCL11A and MCF-7R-si-NC cells. (**B**) Representative western blot showing BCL11A protein expression in MCF-7R-si-BCL11A and MCF-7R-si-NC cells. (**C**) Representative western blots showing PI3K, AKT, p-AKT, MEK, p-MEK, ERK, p-ERK protein expression in MCF-7R-si-BCL11A and MCF-7R-si-NC cells. (**D**) CCK-8 assay showing viability of MCF-7R-si-BCL11A and MCF-7R-si-NC cells with or without TAM treatment. Note: *denotes *P* < 0.05 compared to control; MCF-7R-si-BCL11A denotes MCF-7R cells stably transfected with siRNA against BCL11A; MCF-7R-si-NC denotes MCF-7R cells stably transfected with control siRNA.

Next, we transfected siRNA against BCL11A (MCF-7R-si-BCL11A) and downregulated BCL11A mRNA and protein levels (Figures 6A and 6B). MCF-7R-si-BCL11A cells showed reduced PI3K protein levels accompanied by reduced phosphorylation of ERK, MEK and AKT, whereas total ERK, MEK, and AKT levels were unaffected (Figure 6C). MCF-7R-si-BCL11A cells also showed enhanced TAM sensitivity than MCF-7R-si-NC cells when treated with 0.1 µM 4-OH-TAM for 24 h (Figure 6D). These data suggested that BCL11A contributes to TAM resistance by activating the PI3K/AKT and MAPK pathways, which could be reversed by uc.57 overexpression.

## DISCUSSION

LncRNAs play an important role in the resistance of breast cancer to TAM [11, 19]. In this study, we analyzed eight ultra-conserved lncRNAs (uc.26, uc.41, uc.44, uc.48, uc.51, uc.57, uc.64, uc.250) in the TAM-resistant breast cancer cell line, MCF-7R and its parental cell line, MCF-7. Uc.57 levels were lower in MCF-7R cells than in MCF-7 cells. Moreover, uc.57 expression was lower in breast cancer tissues than in precancerous tissues. These results suggested that uc.57 was associated with breast cancer and TAM resistance. To explore the relationship between uc.57 and TAM resistance further, we searched the UCSC genome database and identified *BCL11A*, which is located on human chromosome 2, as the target gene of uc.57. BCL11A was overexpressed in breast cancer tissues than in precancerous tissues. This was consistent with a previous report that BCL11A promoted triple-negative breast cancer [23]. We observed higher BCL11A mRNA and protein levels in MCF-7R cells than in MCF-7 cells. This suggested that the regulation of BCL11A by uc.57 determined TAM resistance in MCF-7R cells.

SK inhibits estrogen-dependent cell growth and enhances the anti-estrogen activity during endocrine therapy through ER signaling [8]. SK also enhances the therapeutic efficacy of taxol in breast cancer by inhibiting AKT and ERK [24]. PI3K/AKT and MAPK/ERK signaling pathways are important mechanisms of TAM resistance [11–13]. Therefore, we postulated that SK may be beneficial to overcome TAM resistance. The results of the CCK8 assay showed that in MCF-7 cells, both TAM and SK could inhibit the growth of MCF-7R cells and co-treatment of them can be more effective. It is consist with reported literature [8]. In MCF-7R cells, TAM could not inhibit cell growth, whereas SK could. What’s more, the SK and TAM co-treatment exerted the most significant inhibitory effect on MCF-7R proliferation. According to our results, SK+TAM treatment had similar inhibitory effect on MCF-7R and MCF-7 cell growth, which was about twice as much as the inhibitory effect of TAM treatment on MCF-7 cell growth. Moreover, in the human breast tissue-derived mouse models, we found that SK alone and TAM+SK treatments resulted in decreased tumor growth whereas TAM treatment resulted in similar tumor size comparable to the control group. Since single TAM treatment could not inhibit MCF-7R cell growth, the distinctive inhibitory effect of TAM+SK suggested that SK could not only directly inhibit the growth of TAM-resistant breast cancer cells but also decrease TAM resistance in TAM-resistant breast cancer cells. In our following study, we found that the efficacy of SK to overcome TAM resistance in MCF-7R cells was dose-dependent, only if SK was above the threshold concentration. The direct inhibitory effect of SK on MCF-7R cells and the reducing effect of SK on TAM exist at the same time. When SK does not inhibit cell growth, it would not decrease TAM resistance either. It suggests that both two effects work through similar mechanisms. In addition to inhibiting cell growth, SK can induce apoptosis and inhibit metastasis of tumor cells and PI3K/AKT and MAPK/ERK pathways are major mechanisms of such effects [7, 25]. Since TAM resistance is strongly connected with these pathways, we postulated that their inhibition by SK played a significant role in overcoming TAM resistance.

SK treatment resulted in a dose-dependent increase in uc.57 levels in MCF-7R cells. Uc.57 was overexpressed and BCL11A was downregulated in MCF-7R cells treated with TAM+SK or SK alone, whereas control and TAM treated MCF-7R cells showed high BCL11A and low uc.57 expression. Moreover, TAM+SK treated cells showed highest uc.57 expression and lowest BCL11A expression among all groups. In addition to siRNA technology, some diazobenzene-related compounds and methylation inhibitors can modulate lncRNAs in their particular ways [26]. However, natural products and their derivatives related with lncRNAs have not yet been extensively studied. As one of the derivatives, SK was reported to inhibit inflammatory response in rheumatoid arthritis synovial fibroblasts by targeting lncRNA-NR024118 [27], but its association with lncRNAs has not been reported in cancer research. On the other hand, although lncRNAs related to TAM resistance have been previously studied [18, 19], TAM sensitizing agents that target lncRNAs in cancer cells have not been reported. The findings of our study suggest that SK targets uc.57/BCL11A to overcome TAM resistance in breast cancer cells. The PI3K/AKT and MAPK signaling pathways are related to TAM resistance as well as SK function [7, 11]. Both, SK and TAM+SK treatments inhibited the PI3K/AKT and MAPK signaling pathways in addition to suppressing BCL11A expression and the effect of TAM+SK treatment is more significant. These results implicated BCL11A in the SK-mediated attenuation of TAM resistance in MCF-7R cells. Since lncRNAs are associated with cancer growth and progression by cancer pathways [28], activity of PI3K/AKT and MAPK signaling pathways in TAM resistance is mediated by uc.57/BCL11A.

FISH analysis as well as RIP assays demonstrated that uc.57 and BCL11A bind to each other. The MCF-7R-lv-uc.57 cell line overexpressing uc.57 showed downregulation of BCL11A mRNA and protein. Moreover, MCF-7R-lv-uc.57 cells were TAM sensitive, both *in vitro* and *in vivo*. This further confirmed the relationship between uc.57 and TAM resistance.

BCL11A has a critical role in breast cancer stem and progenitor cells [23]. BCL11A knockdown inhibited PI3K/AKT and MAPK pathways in MCF-7R-si-BCL11A cells, thereby sensitizing them to TAM treatment. The activation of PI3K and MAPK pathways directly induces TAM resistance while they are mediated by upstream regulation element, such as IGFR [11, 13]. The data in our study suggested that BCL11A is positively correlated with TAM resistance, and PI3K, MAPK pathways are downstream mechanisms of it.

Our study has several limitations. First, our study lacked clinical evidence because TAM-resistant specimens were not readily available. Second, the detailed mechanism of the regulation of BCL11A by uc.57 and the downstream pathways needs to be explored further. Third, we did not determine if SK decreased TAM resistance in uc.57 KO cells because uc.57 levels were very low. Finally, clinical application of SK requires more preclinical data.

In conclusion, our study demonstrates that SK treatment can decrease the resistance of breast cancer cells to TAM by upregulating uc.57. Uc.57 binds to BCL11A mRNA and suppresses its expression, which results in suppression of TAM-resistant PI3K/AKT and MAPK signaling pathways. Therefore, uc.57 and BCL11A are potential therapeutic biomarkers in TAM-resistant breast cancer patients.

## MATERIALS AND METHODS

### Patient tissue samples

Thirty pairs of breast cancer specimens were acquired from patients that had breast cancer surgery at the First Affiliated Hospital of Nanjing Medical University, China. All tissues were frozen in liquid nitrogen immediately after surgical removal and stored at −80°C. The study was approved by the ethics and research committee of the First Affiliated Hospital of Nanjing Medical University. Informed consent was obtained from all patients included in the study.

### Cell culture and chemicals

The human breast cancer cell line MCF-7 was obtained from the American Tissue Culture Collection (Manassas, VA, USA). The TAM-resistant human breast cancer cell line MCF-7R was kindly provided by Dr. Stephen Ethier (University of Michigan, Ann Arbor, MI, USA). Both cell lines were cultured in Dulbecco’s modified Eagle’s medium (Thermo Fisher Scientific, Inc.) supplemented with 10% fetal bovine serum (Thermo Fisher Scientific, Inc.) and 1% penicillin–streptomycin solution (Thermo Fisher Scientific, Inc.) in a humidified chamber maintained with 5% CO_2_ at 37°C. SK and 4-trans-hydroxytamoxifen (4-OH-TAM, TAM) were obtained from Sigma (Louis, MO, USA).

### CCK-8 cell viability assay

Cell viability was tested by Cell Counting Kit-8 (Beyotime, Shanghai, China) assay in accordance with the manufacturer’s instructions. 5 × 10^3^ cells were seeded into each well of a 96-well plate and incubated for 24 h followed by various chemical treatments. After 24 h, 10 μl of CCK8 solution was added to each well and incubated for 2 h. Then, the plates were read in an automated microplate reader (Tecan, Grodig, Austria) at a wavelength of 450 nm (OD value). Each experiment was performed four times.

### Quantitative real-time polymerase chain reaction (qRT-PCR)

Total RNA was extracted from cells and tissues with Trizol (Invitrogen, Carlsbad, CA, USA) in accordance with the manufacturer’s instructions. Real-time polymerase chain reaction (RT-PCR) was performed with SYBR-green PCR Master Mix (Roche, USA) in a Fast Real-time PCR 7500 System (Applied Biosystems). The primers used were as follows:

GAPDH (forward: 5′-CATGAGAAGTATGACAACAGCCT-3′; reverse: 5′-AGTCCTTCCACGATACCAAAGT-3′); uc.57 (forward: 5′-TGGAAAGTACCACATACCATTGTC-3′; reverse: 5′-AGGGCTCAGACAGCTACTGC-3′); BCL11A (forward: 5′-CCCGCAGGGTATTTGTAAAG-3′; reverse: 5′-GACTTCCGTGTTCGCTTTCT-3′).

GAPDH was used as internal control. The relative expression of uc.57 and BCL11A was calculated by 2^−ΔΔCT^ (ΔCt = Ct value of uc.57 or BCL11A minus Ct value of GAPDH; ΔΔCt = ΔCt of the treatment group minus ΔCt of the treatment group). The experiments were performed in triplicate.

### RNA immunoprecipitation (RIP) assay

RIP assay was performed according to previously published protocol [29] using Mgna RIP Kit (Milipore, Temecula, CA, USA) . Briefly, MCF-7R cells were washed with cold PBS, scraped from plates, and lysed in RIP lysis buffer. The beads and antibody complexes (with IgG or BCL11A antibody) were prepared overnight. The RNAs were immunoprecipitated with antibodies, extracted with Trizol and analyzed by qRT-PCR. Relative amounts of immunoprecipitated RNAs were normalized to the input and plotted as fold enrichment versus the IgG control.

### Western blotting

Total protein lysates were prepared from the various groups of cells and their concentrations were measured with the BCA protein assay kit (ThermoScientific, Tewksbury, MA, USA). Equal amounts of protein samples (60 μg) were separated by 10% SDS-PAGE and transferred onto PVDF membranes (Bio-Rad, CA, USA) for 1 hour. The membranes were blocked in 5% skimmed milk in 1× TBST for 1 h and then incubated overnight with the following primary antibodies according to manufacturer’s instructions: anti-PI3K, anti-AKT, anti-phospho-AKT, anti-ERK, anti-phospho-ERK, anti-MEK and anti-phospho-MEK antibodies were all obtained from Cell Signaling Technology (Danvers, MA, USA); anti-GAPDH antibody was from Bioworld (Nanjing, China), and anti-BCL11A antibody was from Abcam (Cambridge, USA). Then, the membranes were incubated for 2 h with horseradish peroxidase conjugated anti-rabbit and anti-mouse secondary antibodies (Bioworld, Nanjing, China). The blots were developed with the SuperSignal West Femto Maximum sensitivity substrate kit (Thermo Scientific) and the chemiluminiscent signals were detected by the FluorChem E System (ProteinSimple, San Jose, CA, USA). The density of each band was quantified using Image Pro Plus 6 software. The phosphorylated protein expression was normalized to the corresponding total protein levels. The expression of other target proteins was normalized to GAPDH levels.

### Fluorescence *in situ* hybridization (FISH)

MCF-7R cells were fixed in 4% para formaldehyde (Sigma) in PBS (pH 7.4) for 15 min and permeabilized in 0.2% Triton X-100 on ice for 5 minutes. The cells were rinsed with 4× SSC prior to hybridization. Hybridization was carried out at 45°C for 12–14 hours using *in vitro* synthesized uc.57-labeled or BCL11A-labeled sense and antisense RNA probes dissolved in hybridization buffer (50% deionized formamide, 4× SSC, 1× Denhardt’s solution, 10% dextran sulfate, and 20 μg tRNA). The extra probe was removed by successive washing with 2× and 0.5× SSC.

### Cell transfection

Since MCF-7R cells showed low expression of uc.57 and high expression of BCL11A, uc.57 was overexpressed, whereas BCL11A was knocked down in this study. The lncRNA uc.57 was overexpressed in MCF-7R cells with commercial lentiviral constructs (Genepharma, Shanghai, China) to establish a new stable cell line, MCF-7R-lv-uc.57. The MCF-7R cells transfected with LV3 empty construct, MC7–7R-lv-NC was used as a negative control. The MCF-7R cells that were 40% to 50% confluent were infected with lentiviruses with the NC and uc.57 vectors at a multiplicity of 8. Next, 5 μg/ml polybrene (Genepharma, Shanghai, China) was added to the cells to increase transfection efficiency. Stable cell lines were selected in medium containing 3 μg/ml puromycin (Sigma, USA) for 1 week and analyzed for uc.57 expression. To generate BCL11A knockdown MCF-7R cells, siRNA targeting BCL11A (Genepharma, Shanghai, China) was transfected in accordance with the manufacturer’s instructions. MCF-7R cells transfected with control scramble siRNA was used as control.

### Tumor xenograft and tumorigenicity assay

Since MCF-7 cells are estrogen dependent, tumors were established in a novel SCID mice model with human mammary transplant tissue according to previously established protocol [30]. Five-to-seven-week-old female SCID mice (C B-17IcrCrl-scid-bgBR) were purchased from the Model Animal Research Center of Nanjing University (Nanjing, Jiangsu Province, China). Normal fresh discarded human breast tissues were obtained from elective reduction mammoplasty surgery. All protocols were conducted in accordance with the ethical guidelines of the Declaration of Helsinki and were approved by the ethics and research committee of the First Affiliated Hospital of Nanjing Medical University. Three pieces of 4mm^3^ fresh breast tissue were transplanted under the skin on the left mid-dorsal flank of each SCID mouse. After one week, the implanted breast tissues were inoculated with GFP expressing MCF-7R-lv-uc.57 or MCF-7R-lv-NC cells (5 × 10^5^ in 0.2 ml PBS).

Twenty SCID mice were injected with MCF-7R-lv-NC cells into human breast tissue transplants. A week later, they were randomly and equally divided into the control, TAM, SK, and TAM+SK groups and injected every alternate day with either DMSO, 20 μg/ml 4-OH-TAM, 200 μg/ml SK or 200 μg/ml SK plus 20 μg/ml 4-OH-TAM (1 ml each), respectively.

To explore the function of uc.57 in TAM resistance, we transplanted five mice with MCF-7R-con cells and another five mice with MCF-7R-lv-uc.57 cells. Both groups were treated every day with 1ml 20 μg/ml 4-OH-TAM. All the mice were sacrificed and observed grossly by whole body imaging (Illumatool 9900, Lightools Research, Encinitas; CA, USA) to determine the nature of tumors formed. Tumors were measured every week by vernier calipers, and the mice were euthanized after seven weeks. The volume of the implanted tumors was calculated by using the formula, volume = (width^2^ × length)/2. The initial volume of human breast tissue was subtracted from the final volume. The animal experiments were approved by the NJMU Institutional Animal Care and Use Committee.

### Statistical analysis

IBM SPSS Statistics v19 software was used for all statistical analyses. Student’s *t*-test was used to determine significant differences between two groups. Unpaired *t*-test was performed to analyze qRT-PCR data for breast cancer tissues and para-cancerous tissues. A *P* < 0.05 was considered statistically significant.

## References

[1] Siegel RL, Miller KD, Jemal A (2017). Cancer Statistics, 2017. CA: A Cancer Journal for Clinicians.

[2] Early Breast Cancer Trialists' Collaborative G (1998). Tamoxifen for early breast cancer: an overview of the randomised trials. The Lancet.

[3] Ring A, Dowsett M (2004). Mechanisms of tamoxifen resistance. Endocrine-related cancer.

[4] Lu L, Qin A, Huang H, Zhou P, Zhang C, Liu N, Li S, Wen G, Zhang C, Dong W, Wang X, Dou QP, Liu J (2011). Shikonin extracted from medicinal Chinese herbs exerts anti-inflammatory effect via proteasome inhibition. European Journal of Pharmacology.

[5] Chen X, Yang L, Oppenheim JJ, Howard MZ (2002). Cellular pharmacology studies of shikonin derivatives. Phytotherapy Research.

[6] Liang W, Cai A, Chen G, Xi H, Wu X, Cui J, Zhang K, Zhao X, Yu J, Wei B, Chen L (2016). Shikonin induces mitochondria-mediated apoptosis and enhances chemotherapeutic sensitivity of gastric cancer through reactive oxygen species. Scientific Reports.

[7] Lu D, Qian J, Li W, Feng Q, Pan SH, Zhang S (2015). β-hydroxyisovaleryl-shikonin induces human cervical cancer cell apoptosis via PI3K/AKT/mTOR signaling. Oncology Letters.

[8] Yao Y, Zhou Q (2010). A novel antiestrogen agent Shikonin inhibits estrogen-dependent gene transcription in human breast cancer cells. Breast Cancer Research and Treatment.

[9] Zhang Y, Qian RQ, Li PP (2009). Shikonin, an ingredient of Lithospermum erythrorhizon, down-regulates the expression of steroid sulfatase genes in breast cancer cells. Cancer Letters.

[10] Qiaoli Z, Assimopoulou NA, Sabine MK, Harilaos D, Ioanna C, Nadine Kr, José-Luis R, Vassilios P, Rudolf B, Thomas E (2015). Inhibition of c-MYC with involvement of ERK/JNK/MAPK and AKT pathways as a novel mechanism for shikonin and its derivatives in killing leukemia cells. Oncotarget.

[11] Hayes EL, Lewis-Wambi JS (2015). Mechanisms of endocrine resistance in breast cancer: an overview of the proposed roles of noncoding RNA. Breast Cancer Research.

[12] Musgrove EA, Sutherland RL (2009). Biological determinants of endocrine resistance in breast cancer. Nature Reviews Cancer.

[13] Zhang Y, Moerkens M, Ramaiahgari S, de Bont H, Price L, Meerman J, van de Water B (2011). Elevated insulin-like growth factor 1 receptor signaling induces antiestrogen resistance through the MAPK/ERK and PI3K/Akt signaling routes. Breast Cancer Research.

[14] Chen X, Zhao M, Hao M, Sun X, Wang J, Mao Y, Zu L, Liu J, Shen Y, Wang J, Shen K (2013). Dual inhibition of PI3K and mTOR mitigates compensatory AKT activation and improves tamoxifen response in breast cancer. Molecular Cancer Research.

[15] Ghayad SE, Vendrell JA, Ben Larbi S, Dumontet C, Bieche I, Cohen PA (2009). Endocrine resistance associated with activated ErbB system in breast cancer cells is reversed by inhibiting MAPK or PI3K/Akt signaling pathways. International Journal of Cancer.

[16] Shah KN, Mehta KR, PetersonD, Evangelista M, Livesey JC, Faridi JS (2014). AKT-induced tamoxifen resistance is overturned by RRM2 inhibition. Molecular Cancer Research.

[17] Yang G, Lu X, Yuan L (2014). LncRNA: A link between RNA and cancer. Biochimica et Biophysica Acta.

[18] Godinho MFE, Sieuwerts AM, Look MP, Meijer D, Foekens JA, Dorssers LCJ, Van-Agthoven T (2010). Relevance of BCAR4 in tamoxifen resistance and tumour aggressiveness of human breast cancer. British Journal of Cancer.

[19] Xue X, Yang YA, Zhang A, Fong KW, Kim J, Song B, Li S, Zhao JC, Yu J (2016). LncRNA HOTAIR enhances ER signaling and confers tamoxifen resistance in breast cancer. Oncogene.

[20] Bejerano G PM, Makunin I, Stephen S, Kent WJ, Mattick JS, Haussler D (2004). Ultraconserved elements in the human genome. Science.

[21] Katzman S KA, Bejerano G, Fewell G, Fulton L, Wilson RK, Salama SR, Haussler D (2007). Human genome ultraconserved elements are ultraselected. Science.

[22] Peng JC, Shen J, Ran ZH (2013). Transcribed ultraconserved region in human cancers. RNA Biology.

[23] Khaled WT, Choon Lee S, Stingl J, Chen X, Raza Ali H, Rueda OM, Hadi F, Wang J, Yu Y, Chin SF, Stratton M, Futreal A, Jenkins NA (2015). BCL11A is a triple-negative breast cancer gene with critical functions in stem and progenitor cells. Nature Communications.

[24] Li W, Liu J, Jackson K, Shi R, Zhao Y (2014). Sensitizing the therapeutic efficacy of taxol with shikonin in human breast cancer cells. Plos One.

[25] Chen Y, Zheng L, Liu J, Zhou Z, Cao X, Lv X, Chen F (2014). Shikonin inhibits prostate cancer cells metastasis by reducing matrix metalloproteinase-2/-9 expression via AKT/mTOR and ROS/ERK1/2 pathways. International immunopharmacology.

[26] Tang JY, Lee JC, Chang YT, Hou MF, Huang HW, Liaw CC, Chang HW (2013). Long noncoding RNAs-related diseases, cancers, and drugs. The Scientific World Journal.

[27] Yang KY, Chen DL (2015). Shikonin Inhibits Inflammatory Response in Rheumatoid Arthritis Synovial Fibroblasts via lncRNA-NR024118. Evid Based Complement Alternat Med.

[28] Schmitt Adam M, Chang Howard Y (2017). Long Noncoding RNAs in Cancer Pathways. Cancer Cell.

[29] Tsai MC, Manor O, Wan Y, Mosammaparast N, Wang JK, Lan F, Shi Y, Segal E, Chang HY (2010). Long noncoding RNA as modular scaffold of histone modification complexes. Science.

[30] Wang J, Xia TS, Liu XA, Ding Q, Du Q, Yin H, Wang S (2010). A novel orthotopic and metastatic mouse model of breast cancer in human mammary microenvironment. Breast cancer research and treatment.

